# Virtual Sonography Through the Internet: Volume Compression Issues

**DOI:** 10.2196/jmir.3.2.e21

**Published:** 2001-06-22

**Authors:** Olga Ferrer-Roca, Joseba Vilarchao-Cavia, Juan-Mario Troyano-Luque, Matilde Clavijo

**Affiliations:** ^1^Chair of Pathology and UNESCO Chair of TelemedicineUniversity of La LagunaSpain; ^2^Fellow at the UNESCO Chair of TelemedicineSpain; ^3^Chief of the Gynecology Ultrasound Unit. Clinic HospitalTenerifeSpain; ^4^Assistant doctor at the Ultrasound Unit. Clinic HospitalTenerifeSpain

**Keywords:** Virtual sonography, telemedicine, 3D-ultrasound, 3-D ultrasound, obstetrics, volume rendering

## Abstract

**Background:**

Three-dimensional ultrasound images allow virtual sonography even at a distance. However, the size of final 3-D files limits their transmission through slow networks such as the Internet.

**Objective:**

To analyze compression techniques that transform ultrasound images into small 3-D volumes that can be transmitted through the Internet without loss of relevant medical information.

**Methods:**

Samples were selected from ultrasound examinations performed during, 1999-2000, in the Obstetrics and Gynecology Department at the University Hospital in La Laguna, Canary Islands, Spain. The conventional ultrasound video output was recorded at 25 fps (frames per second) on a PC, producing 100- to 120-MB files (for from 500 to 550 frames). Processing to obtain 3-D images progressively reduced file size.

**Results:**

The original frames passed through different compression stages: selecting the region of interest, rendering techniques, and compression for storage. Final 3-D volumes reached 1:25 compression rates (1.5- to 2-MB files). Those volumes need 7 to 8 minutes to be transmitted through the Internet at a mean data throughput of 6.6 Kbytes per second. At the receiving site, virtual sonography is possible using orthogonal projections or oblique cuts.

**Conclusions:**

Modern volume-rendering techniques allowed distant virtual sonography through the Internet. This is the result of their efficient data compression that maintains its attractiveness as a main criterion for distant diagnosis.

## Introduction

Image-communication systems for medical images have bandwidth (data-transfer capacity) and image-size constraints that result in time-consuming connections for uncompressed raw-image data. Image compression is a key factor to improving transmission speed and storage, but it risks losing relevant medical information.

The radiology standard DICOM3 (Digital Imaging and Communications in Medicine, Version 3.0) [1] provides rules for compression using lossless JPEG (Joint Photographic Expert Group) methods. However, there are no rules for acceptance of lossy compression in medical imaging-it is an extremely subjective decision. Acceptable levels of compression should never sacrifice diagnostic information.

Ultrasonography has always been envisaged as one of the easiest telemedicine applications due to the small size of images with a dynamic range of 8 bits [2]. A new era of daily patient-care, even at a remote site, is expected using volume-rendering techniques for 3-D reconstruction of noisy ultrasound (ultrasound) images.

The three most common radiology reconstruction techniques are: shaded surface display, maximum intensity projection for x-ray simulation [3], and 3-D volume rendering for solid 3-D reconstruction [4]. In 3-D volume rendering, volume data management includes special techniques for acquisition (in our case moving the ultrasound probe by hand), re-sampling (particularly detailed because of the compression it achieved) and editing the data set by "flying-through", "flying around", multiple-view display, obscured structure and shading depth cues, or kinetic and stereo depth cues [5].

In 3-D reconstructions, the original ultrasound "moving frames" are composed of 500 to 550 single frames of 512-pixel x 512-pixel-spatial resolution, and the size of the final image (100- to 120-MB) is too large to be sent through the Internet. This paper presents the experience of our team on 3-D ultrasound, focusing on data-reduction techniques that allow teleconsultation through the Internet.

## Methods

This trial used ultrasound examinations carried out in the ultrasound unit in the Obstetrics and Gynecology Department at the University Hospital in La Laguna, Tenerife, Canary Islands, Spain in 1999 and 2000.

The ultrasound equipment used was an Aloka-SSD 680 ultrasound device connected to a PC. Acquisition was carried out by moving the ultrasound probe by hand. The probe was a 5-MHz curvilinear abdominal probe transducer, attached to a magnetic-field positioning device. The probe position, with 6 degrees of freedom, was transmitted to the computer by an ISA (Industry Standard Architecture) PC-Bird board,. A Falcon digitizing board captured frames with 8-bit dynamic range.

Our PC (Personal Computer) was a 450-MHz dual Pentium II computer, with 256-MB RAM using a Windows NT operating system.

Volume rendering and display were carried out with the TeleInVivo ™ volume visualization software (Fraunhofer Center for Research in Computer Graphics, Darmstadt, Germany) commercialized by MedCom ™. Three builds of version 3.3 of the software were tested: build 1400, build 1500, and build 1510.

The PC was connected to the Internet through a standard 100bT (also known as a 100BaseT) LAN (Local Area Network) board. Images were transmitted using TCP/IP (Transmission Control Protocol/Internet Protocol ) through the Internet, either to other countries (eg, Coimbra in Portugal) or to smaller islands (eg, La Palma, Canary Islands).

**Figure 1:**
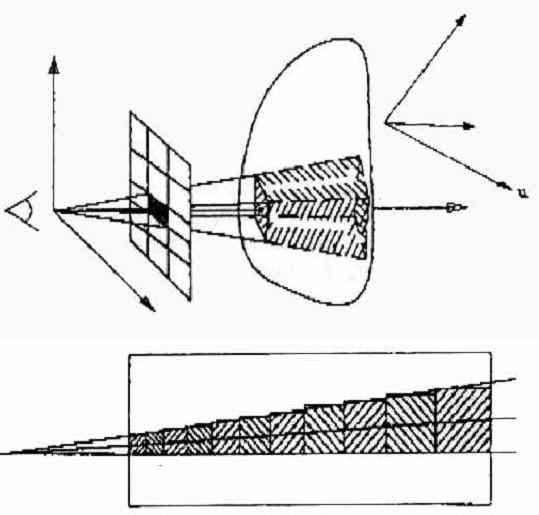
Detail of the pyramid casting technique. The vertex is the eye-view. The rendering allows distance sampling to be expressed as resolution (algorithm 1, top drawing) or the volume size of the final voxel (algorithm 2, bottom drawing). For algorithm 1 the voxel volume is 100%. Image taken with permission of the author G. Sakas [8] and of the editor of The Visual Computer

The acquired original moving frames with 8-bit dynamic range were resampled and then converted into a volume data set that combined frames together with their position and orientation into a single 3-D image, with a system accuracy less than 1 mm.

Resampling is a geometrical transformation of the ultrasound-pixels into the 3-D-voxel (volume element) spaces based on the tracking measurements. Resampling transforms the 2-D sequential images into a single volume data set and is carried out in the TeleInVivo ™ software using the pyramid-casting technique [6,7]. The pyramid-casting technique is a modification of ray-casting that improves rendering speed by reducing distance sampling and averaging pyramidal voxels (Figure 1, from [7]) to produce "cloud" representation of the 3-D ultrasound image.

Data editing used the well-known maximum-intensity and minimum-intensity projections, x-ray absorption, and surface visualization by gradient or cloud applied in the pyramid-casting algorithm. The "flying around" technique, which can be recorded with video, is currently used for display. The TeleInVivo ™ software package allowed us to see 2-D orthogonal cuts of the 3-D reconstructed volume and allowed us to obtain oblique cuts from the volume, allowing virtual sonography, which is available after transmission-even at a remote site.

To illustrate the image quality and compression techniques obtained by the software, we chose 505 digitized frames (slices) of a fetus with an encephalocele. In Results we show 4 sets of an orthogonal plane (slice 86, equivalent to a 2-D-ultrasound image view) together with a 3-D volume reconstruction. The volume was displayed with the maximum intensity projection algorithm. Selected parameters were: a contrast of -0.34 and an intensity of 1.03; surface mixing of 45% with a semitransparent surface algorithm having a mean gray value of 157 and a tolerance of 169; depth weight of 0; contrast of -0.36; and intensity of 1.13. The surface was displayed with high quality and medium smoothing.

## Results

**Figure 2:**
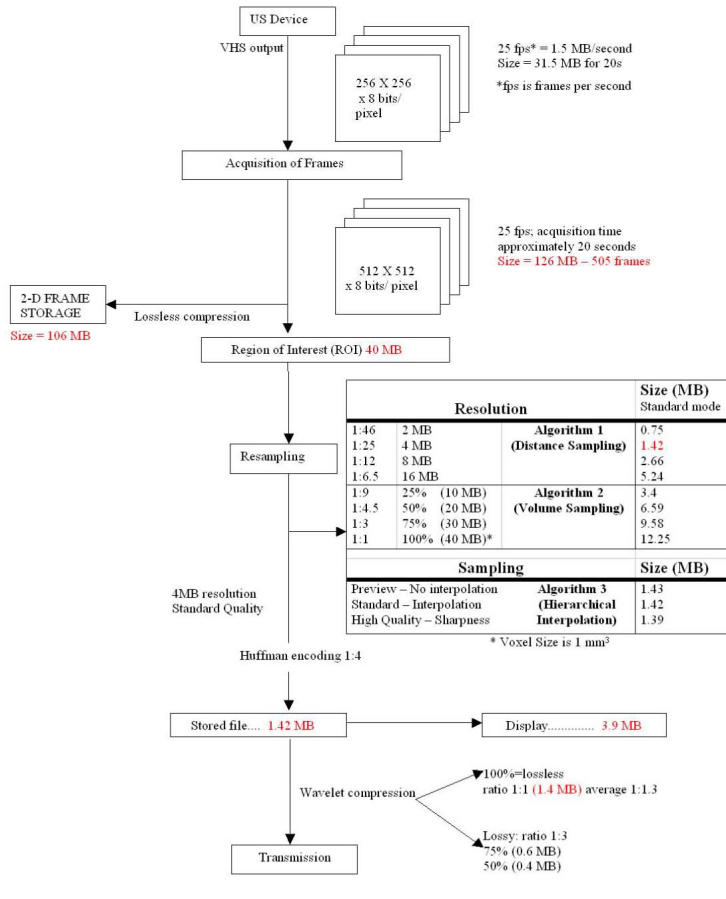
Compression scheme of the TeleInVivo software. MB data indicates file size

The moving frames, recorded with the ultrasound device, have an original size of 100 to 120 MB (from 500 to 550 single frames). This size must be drastically reduced. The size-reduction process is shown in Figure 2, together with intermediate file sizes obtained using an example with an original size of 126 MB before storage and 106 MB after storage.

### First Step: ROI (Region of Interest) selection

The parts of the moving frame with no relevant clinical information such as background and/or non-interesting parts are deleted. In this step original images of 106 MB and 505 frames were reduced to 40 MB (62% reduction).

### Second Step: volume rendering (Resolution/Sampling portion of figure 2)

Volume rendering transforms the original data into a collection of visible primitives (basic shapes) from the 3-D object, which can be viewed from any direction in space. Resampling was carried out with a pyramid casting technique that selects the resolution and the degree of interpolation required for visualization based on the sampling quality.

**Figure 3:**
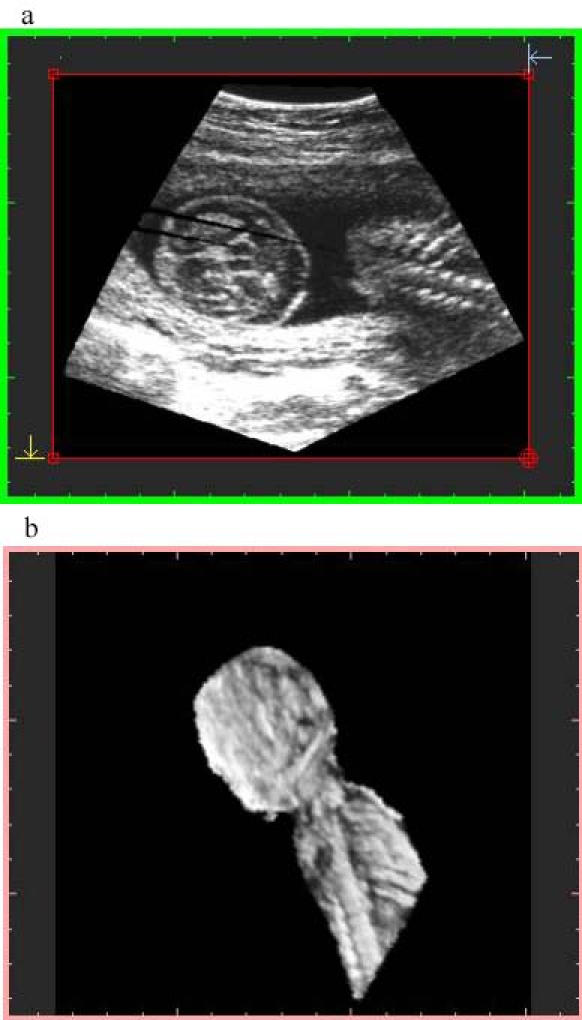
Image generated with algorithm 2 at 75% resolution and high-quality sampling. File size is 30 MB at display and 9.63 MB stored. Top image: orthogonal plane slice 86s. Notice the gaps on the left. Bottom image: 3-D image

**Figure 4:**
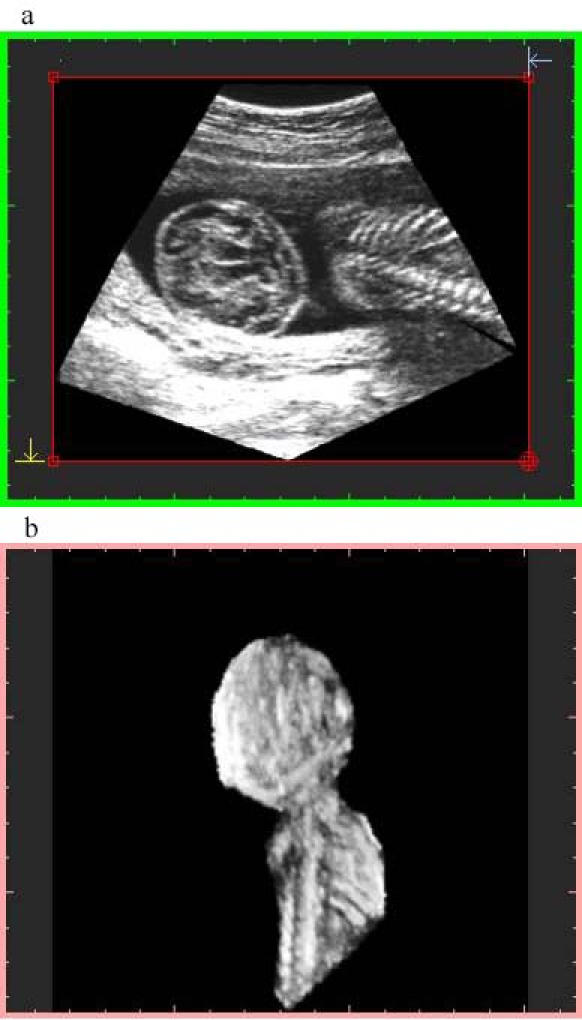
Image generated with algorithm 2 at 75% resolution and preview sampling. File size is 30 MB at display and 9.25 MB stored. Top image: orthogonal plane slice 86s. Notice the gap on the right. Bottom image: 3-D image

Resolution can be chosen by means of 2 algorithms:

- Algorithm 1 takes into account the memory space of the geometry buffer and selects the "distance sampling" required for it, resulting in data sets of 16 MB (about 256^3^), 4 MB (about 160 ^3^), and 2 MB (about 128^3^).
- Algorithm 2 considers the size of the voxel that is averaged, using a "pyramidal volume" method for sampling. At 100% resolution, the size reached the system resolution (1 mm^3^); lower percentages give rise to bigger voxels introducing gaps on the orthogonal plane images that were not visible on the volume data. A detail of a gap using a 75% voxel size is shown in Figure 3 and Figure 4. The size and frequency of these gaps increased when lower percentages were chosen, resulting in orthogonal images that did not produce a proper diagnosis.

**Figure 5:**
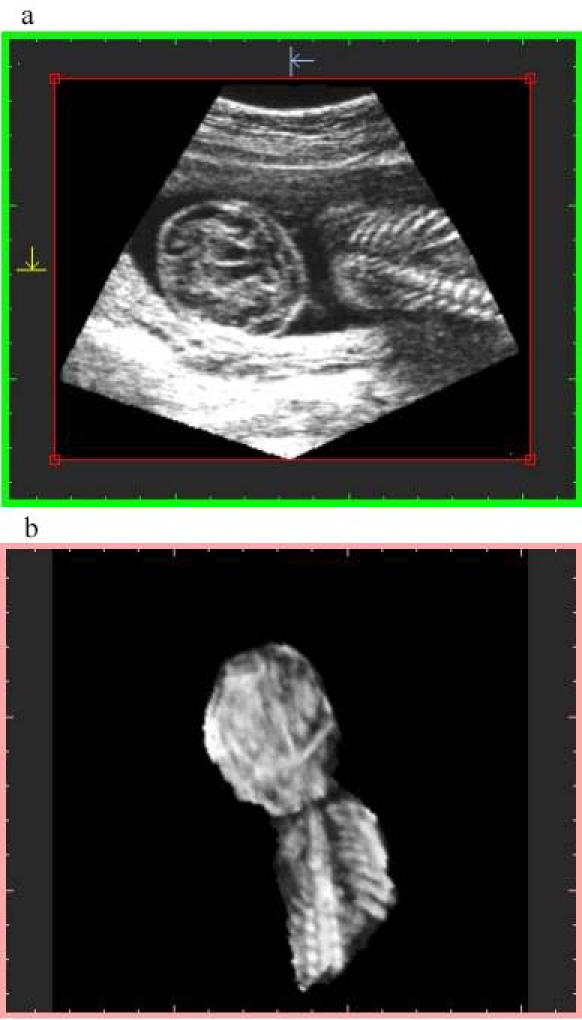
Image at 4-MB resolution with preview sampling. File size is 3.9 MB at display and 1.43 MB stored. Top image: orthogonal plane slice 86. Bottom image: 3-D image

**Figure 6:**
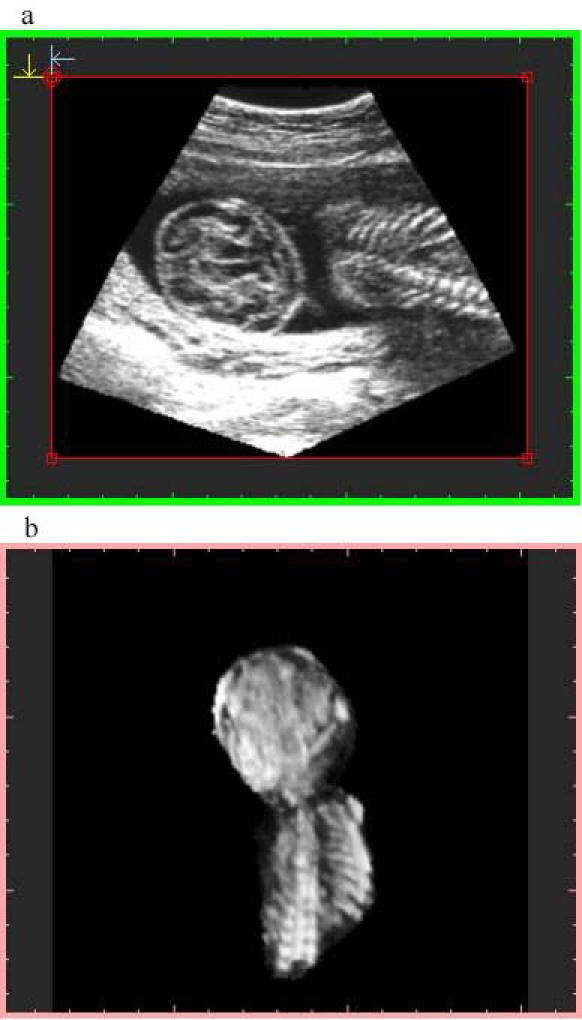
Image at 4-MB resolution with high-quality sampling. File size is 3.9 MB at display and 1.39 MB stored. Top image: orthogonal plane slice 86. Bottom image: 3-D image

**Figure 7:**
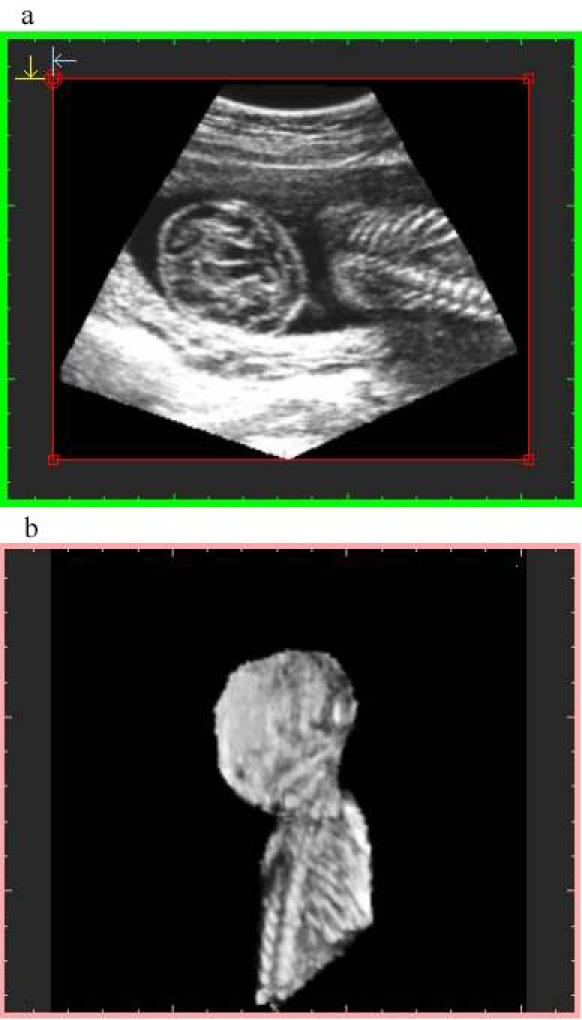
Image at 4-MB resolution with standard- sampling. File size is 3.9 MB at display and 1.42 MB stored. Top image: orthogonal plane slice 86. Bottom image: 3D image

**Figure 8:**
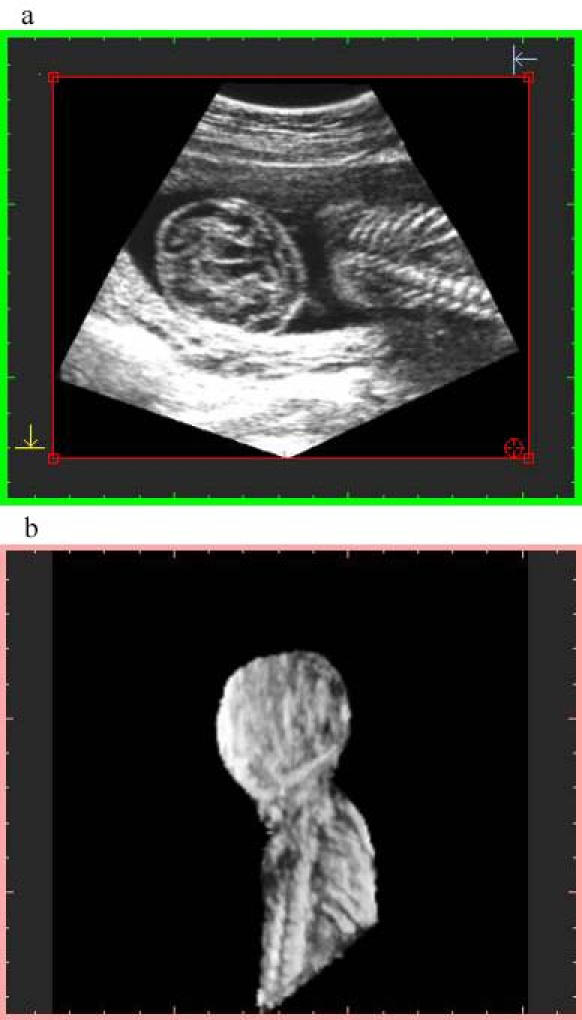
Image at 16-MB resolution with standard sampling. File size is 15.8 MB at display and 5.24 MB stored. Top image: orthogonal plane slice 86. Bottom image: 3D image

Algorithm 2 produces a larger final-file-size and a lower-quality image (Figure 3,Figure 4), due to the voxel averaging technique. Algorithm 1 produces a smaller final-file-size and a higher-quality image (Figure 5,Figure 6,Figure 7,Figure 8).

Sampling quality is based on degree of interpolation and sharpness provided by algorithm 3. It produces a visual representation by hierarchical interpolation of the sampled data obtained with Algorithms 1 and 2, resulting in no interpolation with preview sampling (Figure 4 and Figure 5) and higher-sharpness with high-quality sampling (Figure 3 and Figure 6).

### Third Step: Huffman encoding

When volume data is stored, it is compressed with a Huffman-encoding technique, which is lossless, of about 1:3 compression ratio (Table 1). The size of displayed images and the final size of stored images used for teleconsultation are shown in Table 1. All possible combinations provided by the available rendering algorithms are listed. The compression ratio can vary from image to image; in Figure 2 the compression ratio is 1:4.

**Table 1:** Sizes of displayed and stored images using TeleInVivoTM rendering algorithms. Image quality (attractiveness) can be seen in the Figures listed in the last column

Algorithms	**Memory Display, MB**	**Stored File, MB**	**Huffman compression ratio for Transmission**	Figure
2 MB-High quality	2.0	0.73	1: 2.7	
2 MB-Standard	2.0 MB	0.75	1: 2.6	
2 MB- Preview	2.0	0.76	1: 2.6	
4 MB-High quality	3.9	1.39	1: 2.4	Figure 6
4 MB-Standard	3.9	1.42	1: 2.8	Figure 7
4 MB-Preview	3.9	1.43	1: 2.7	Figure 5
8 MB-High quality	7.8	2.63	1: 3	
8 MB-Standard	7.8	2.66	1: 2.9	
8 MB- Preview	7.8	2.67	1: 2.9	
16 MB-High quality	15.8	5.22	1: 3	
16 MB-Standard	15.8	5.24	1: 3	Figure 8
16 MB-Preview	15.8	5.18	1: 3	
25%-High quality	9.9	3.36	1: 3	
25%-Standard	9.9	3.40	1: 2.9	
25%-Preview	9.9	3.40	1: 2.9	
50%-High quality	20	6.57	1: 3	
50%-Standard	20	6.59	1: 3	
50%-Preview	20	6.44	1: 3.1	
75%-High quality	30	9.63	1: 3.1	Figure 3
75%-Standard	30	9.58	1: 3.1	
75%-Preview	30	9.25	1: 3.2	Figure 4
100%-High quality	39.7	12.38	1: 3.2	
100%-Standard	39.7	12.25	1: 3.2	
100%-Preview	39.7	11.79	1: 3.4	

### Fourth Step: Compression before transmission

Before transmitting the image, two compression techniques were used. The lossy wavelet algorithm produced, in the final file, a 1:3 compression without a significant loss of the visual image quality. Nevertheless image transmission in the present trial was carried out with a lossless technique. Compression achieved in this phase was negligible for 4-MB resolution images (Figure 2). For 16-MB images compression in this phase was 1:2.

To compare lossless compression provided by the software we used with regular lossless compression techniques (such as WinZip compression) a lossless JPEG algorithm was applied to the original frame images. Compression ratios were 1:4 to 1:5.

There were 101 cases that used consultation through the Internet. Only 3 of these cases were resampled at 16-MB resolution. The remaining cases were resampled at 4-MB resolution with algorithm 1. This was done because visual image quality did not show subjective differences (Figure 7 and Figure 8).

During consultations the telecommunication line broke down 17 times. The mean transmission time per image was 6.8 minutes, with an average data throughput of 6.6 Kbytes per second.

**Figure 9:**
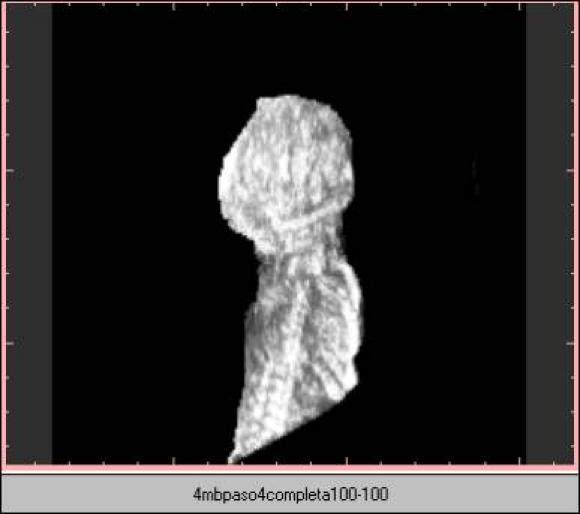
Image at 4-MB resolution with standard sampling. Sine Loop video-image.

**Figure 10:**
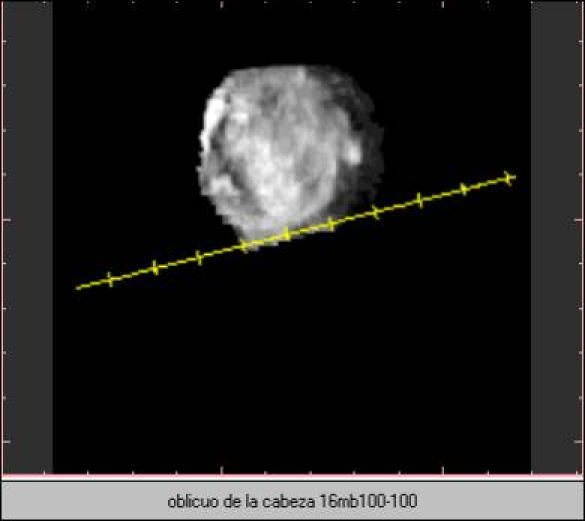
Image at 16-MB resolution with standard sampling.

Distant diagnosis was possible on 94 out of 101 transmitted images. The transmitted images that did not allow distant diagnosis were bad-quality images, due to acquisition difficulties related to moving the probe by hand [9].

The volume-rendering technique makes it possible to cut the volumetric image in all directions. This technique allows "offline" virtual sonography, both locally and at distance, that does not require the patient's presence. Software facilities allow recording volume movement in short videos that help visualization (Figure 9 and Figure 10).

The 2-D orthogonal planes were the ones used by the doctors for diagnostic purposes. The oblique cuts were helpful in only 2 of the 101 cases: a case of an ectopic pregnancy in a rudimentary uterine corn, and an abdominal implant of a pinealoblastoma of the brain that was drained, for treatment purposes, into the abdominal cavity.

## Discussion

The present work summarizes one-year's experience with 3-D-ultrasound image acquisition and processing, using a device that can provide virtual sonography and teleconsultation at distance. The was done with an external add-on system in an existing 2-D ultrasound device, at the sonography unit of the Obstetrics and Gynecology Department, University Hospital of Tenerife, Canary Islands, Spain.

The clinical expectations offered by inexpensive 3-D acquisition systems such as the one presented here are high, particularly because they can be used in "noisy" images, such as sonographic images, and also because they provide teleconsultation facilities. Furthermore, the capability to cut volume data in all spatial directions, producing distant and local virtual sonography [8] improves diagnostic procedures. In the present trial, teleconsultation was possible due to the small size of final 3-D files obtained by volume-rendering techniques.

Our results showed that the essential compression processes were related to the volume-rendering technique and were so efficient that further compression (such as compression before transmission) was unnecessary.

Although final 4-MB files provided an adequate medical visual quality for diagnosis, they did not contain individual pixel data anymore; instead they have "volume primitives" obtained during the pyramid-casting rendering technique at specific sampling frequency. This file is therefore highly optimized for redundancies (that is, due to the type of stored data it is not possible to have any redundant values).

We used compression-before-transmission values as high as 1:2 only for 16-MB-quality images, but those images did not substantially improve the visual perception and had the disadvantage of increased file-size.

These results have two main consequences:

- The degree of compression achieved was very high (1:25 with 4-MB resolution), allowing Internet teleconsultation with 3-D-ultrasound reconstructed images.
- The exact compression technique applied to the medical image was obscure, making it difficult to evaluate, from the medical point of view, whether or not relevant information was lost. Neither the technical manuals from MedCom™ nor the publications of the research team [6,7,9] clarified how the compression achieved by volume rendering using the pyramid-casting technique affects an individual medical image.

According to our previous results [8], the resampling provided by algorithm 1 allowed ultrasound diagnosis at a distance because reconstructed images had the "attractiveness attribute," so that doctors feel comfortable with the esthetic component of the images [2].

Resampling provided by algorithm 2 that caused gaps in the 2-D orthogonal planes, the essential images for diagnosis (since 3-D reconstruction was only used in 2 of the 101 teleconsulted cases), did not have the attractiveness attribute. Although the 100% voxel sampling provided good-quality images, the size of the resulting final volumes was too big for efficient teleconsultation.

Additional problems are: finding out: if algorithm-1 images fulfilled the remaining attributes, such as fidelity and informativeness (an image attribute based on visibility and detectability) [2] for original ultrasound images that are noisy by definition and determining how rendering lossy-compression modifies the visibility and detectability of a specific pathology.

In the present experience, the relatively small size of the final files (1.5 to 2 MB) facilitated the 3-D-ultrasound teleconsultations, even through low-bandwidth networks such as the Internet. Constraints linked to distant reception of static volumes [3] were overcome by virtual sonography, which allowed 2-D cuts in all spatial directions and "sine loop" moving video files.

In summary, volume-rendering techniques applied to ultrasound freehand image acquisition achieved a degree of compression such that teleconsultation through the Internet is possible, but it is still not clear if the rendering techniques could modify visibility and detectability of specific pathologies.

## Abbreviations

ACR/NEMA: American College of Radiology/National Electrical Manufacturers Association
CATAI: Center of Advanced Technology in Image Analysis
DICOM3: Digital Imaging and Communication in Medicine, Version 3.0
EC: European Community
FIS: Fondo de Investigaciones Sanitario
fps: frames per second
IGD: Institut Graphische Datenverarbeitung
IP: Interconnection Protocol
ISA: Industry Standard Architecture
JPEG: Joint Photographic expert group
LAN: Local Area Network
MB: Megabyte
MHz: Megahertz
PC: Personal Computer
RAM: Random Access Memory
ROI: Region of Interest
TCP: Transfer Control Protocol
TCP/IP: Transmission Control Protocol/Internet Protocol
UNESCO: United Nations Educational, Scientific, and Cultural Organization
US: Ultrasound
